# Analysis of the Rock Failure Cone Size Relative to the Group Effect from a Triangular Anchorage System

**DOI:** 10.3390/ma13204657

**Published:** 2020-10-19

**Authors:** Józef Jonak, Robert Karpiński, Michał Siegmund, Andrzej Wójcik, Kamil Jonak

**Affiliations:** 1 Department of Machine Design and Mechatronics, Faculty of Mechanical Engineering, Lublin University of Technology, Nadbystrzycka 36, 20-618 Lublin, Poland; r.karpinski@pollub.pl (R.K.); a.wojcik@pollub.pl (A.W.); 2 KOMAG Institute of Mining Technology, Pszczyńska 37, 44-100 Gliwice, Poland; msiegmund@komag.eu; 3 Department of Clinical Neuropsychiatry, Medical University of Lublin, ul. Gluska 1, 20-439 Lublin, Poland; kamiljonak@umlub.pl; 4 Department of Computer Science, Lublin University of Technology, 20-618 Lublin, Poland

**Keywords:** FEM, fracture mechanics, numerical modeling of fracture, rock mechanics

## Abstract

This study employs the numerical analysis and experimental testing to analyze the fracturing mechanics and the size of rock cones formed in the pull-out of a system of three undercut anchors. The research sets out to broaden the knowledge regarding: (a) the potential of the undercut anchor pull-out process in mining of the rock mass, and (b) estimating the load-carrying capacity of anchors embedded in the rock mass (which is distinctly different from the anchorage to concrete). Undercut anchors are most commonly applied as fasteners of steel components in concrete structures. The new application for undercut anchors postulated in this paper is their use in rock mining in exceptional conditions, such as during mining rescue operations, which for safety considerations may exclude mechanical mining techniques, mining machines, or explosives. The remaining solution is manual rock fracture, whose effectiveness is hard to assess. The key issue in the analyzed aspect is the rock fracture mechanics, which requires in-depth consideration that could provide the assistance in predicting the breakout prism dimensions and the load-displacement behavior of specific anchorage systems, embedment depth, and rock strength parameters. The volume of rock breakout prisms is an interesting factor to study as it is critical to energy consumption and, ultimately, the efficiency of the process. Our investigations are supported by the FEM (Finite Element Method) analysis, and the developed models have been validated by the results from experimental testing performed in a sandstone mine. The findings presented here illuminate the discrepancies between the current technology, test results, and standards that favor anchorage to concrete, particularly in the light of a distinct lack of scientific and industry documentation describing the anchorage systems’ interaction with rock materials, which exhibit high heterogeneity of the internal structure or bedding. The Concrete Capacity Design (CCD) method approximates that the maximum projected radius of the breakout cone on the free surface of concrete corresponds to the length of at the most three embedment depths (*h*_ef_). In rock, the dimensions of the breakout prism are found to exceed the CCD recommendations by 20–33%. The numerical computations have demonstrated that, for the nominal breakout prism angle of approx. 35% (CCD), the critical spacing for which the anchor group effect occurs is ~4.5 (a cross-section through two anchor axes). On average, the observed spacing values were in the range of 3.6–4.0.

## 1. Introduction

The numerous empirical models proposed thus far have described the breakout cone formation mechanism in concrete that accompanies anchor pull-out in common constructions and assembly technologies [1,2]. Elements of the theory describing the load-carrying capacity of anchor systems have been in part developed by the research teams led by Eligehausen [3,4,5,6]. The primary focus of similar studies has been directed towards determining the minimum anchor pull-out force with respect to their predicted load-carrying capacity [7,8,9]. For industrial use, simplified anchorage designing methods have been created for a range of fastener layouts [10,11,12]. The recommendations have been subject to numerous revisions, which aimed to improve the operational safety of anchorages and the design strength forecasting [13]. The question of the trajectory and the range of projected crack propagation in concrete failure is typically regarded as a secondary issue and its product is approximated to a pyramid or a cone (CCD—Concrete Capacity Design method) [3,14]. Brittle concrete failure from insulated anchors takes the form of a concrete prism. The crack is assumed to grow at an angle of 35° from the anchor head towards the surface and corresponds to a wedge or cone, in accordance with CEN/TS 1992-4 [13]. Each form of failure is known to be caused by a different value of the force and takes a different form (volume of the fractured material). The current research trends highlight the interest in the reliability of anchor fastenings under cyclical loading associated with, e.g., earthquakes [15].

Several models for predicting the load-carrying capacity of anchorages (the pull-out force) and the size of failure on the free surface of concrete have been proposed, all of which were rooted in the elastic linear fracture mechanics or non-linear fracture mechanics (e.g., Eligehausen [4], Piccinin [16], Brincker [17], Ballarini [18]). In the study by Kaczmarczyk et al. [19], a computational framework for quasi-static brittle fracture in three-dimensional solids is presented [20,21]. The paper sets out the theoretical basis for determining the initiation and direction of crack growth based on the concept of configurational mechanics, consistent with Griffith’s theory. Problems of crack propagation on the failure surface in continuous mass are also discussed, e.g., by Gordeliy et al. and Vogel et al. [22,23,24].

Due to the substantial brittleness of rock members, attempts have been made to adapt Prof. Rice’s theory [25], e.g., in the analysis of crack propagation in rock fracture by coupled mechanical and hydraulic action, which involved the determination of the breakout force [26]. A special feature of the J-integral is that it is path-independent in an isotropic medium. In linear fracture mechanics, the J-integral is equal to the energy release rate Gf. Considering rock material and its heterogeneity, the J-integral, formulated by Rice, becomes path-dependent. Therefore, given that the rock material extracted from particular mines did exhibit marked heterogeneity, the applicable J-integral variant would be the path-dependent one. It was, thus, resolved that the J-integral would not be incorporated in the analytical part of this work as it would entail additional calculations and further laborious research.

In recent years, the accuracy of anchorage load-carrying capacity forecasting has been significantly improved with the aid of artificial intelligence methods [27].

In a different work, Duan et al. have studied the group effect in the 3D dynamic crack growth with a simplified XFEM algorithm [28].

Experiments have shown that crack propagation is primarily affected by the depth of anchoring, commonly known as the effective embedment depth (*h*_ef_). It has been proven that in shallow embedment depths, the breakout prism angle (relative to the surface perpendicular to the anchor axis) is approximately 28° and increases up to 45° as the anchor is embedded deeper into the material [29]. Crack propagation is extensively discussed by Watson, who used FEM systems in the analytical process and accounted for a range of various anchor designs [30].

**Figure 1:**
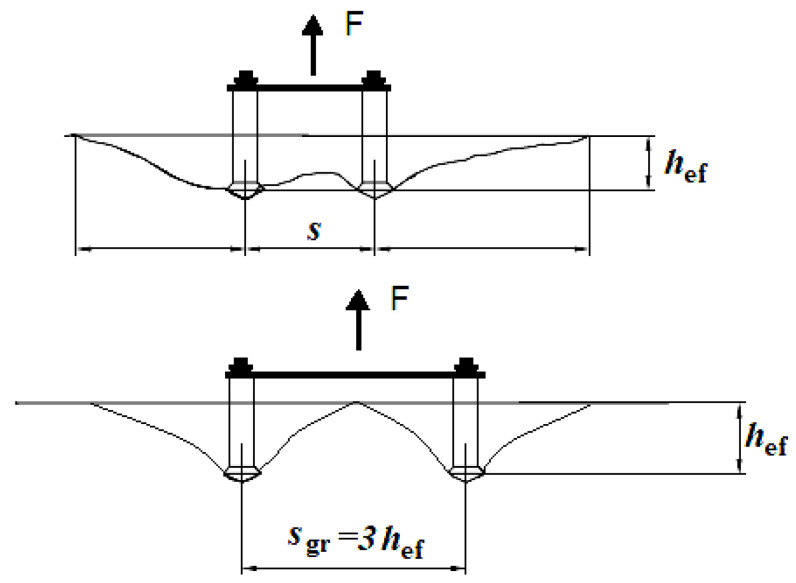
The group effect in cone failure relative to anchor spacing *s*—a system of 2 anchors.

The group effect in cone failure typically occurs in anchorage systems that exceed the critical spacing limit (i.e., *s*_gr_ ~3*h*_ef_, as in Figure 1). Numerous studies have underlined the considerable reduction in the load-carrying capacity of anchor systems, or the drop in the pull-out force [4,18,31,32,33].

In the study of the load capacity and the group effect characteristics of the system of four chemical anchors, Lehr [31] employs MAcroscopic Space Analysis (MASA), i.e., a non-linear 3D FE (Finite Element) program, which is shown to provide good results of correlation between the type of damage and embedment depth. Ballarini et al. [18] modeled linear elastic fracture based on the mechanics of discrete crack propagation for an anchor group in concrete breakout under tension. The anchor group was modeled as a periodic arrangement and the load-carrying capacity and crack propagation were obtained as a function of the relative depth of embedment and anchor spacing.

The ratio between anchor spacing (the smallest distance between the anchor axes) and the effective embedment depth is commonly singled out as a crucial factor to consider in the analysis of the group effect in cone failure [5,18]. Frequently, the research works in the field are computer-aided and utilize the FEM ABAQUS system, which is commonly used to describe failure mechanics [34,35,36,37,38,39]. The load-carrying behavior of multi-fastener anchorage systems and the theoretical mechanism of concrete rupture subject to various complex loading conditions has been explored in the works of Hüer et al. and Tsavdaridisin et al. [40,41].

The problem of group effect in cone failure in a system of two anchors is illustrated in Figure 1.

A marked increase in the general utilization of artificial intelligence techniques (artificial neural networks) that has been observed in experimental science in recent years has been equally notable in the anchorage strength testing domain. In an attempt to predict anchor load-carrying capacity, the input data of the models were modified to test the effect of embedment depth, anchor head diameter, concrete strength, and anchorage systems [27].

Researchers have indicated [42,43] that little is known of the sparsely studied aspects of non-rectangular anchor fastener systems with an odd number of anchors. However, it is important to note that the range of applied anchoring combinations in industry environments is astounding, particularly given the evident paucity of scientific and industry standards describing *non-standard* assemblages, including systems with more than three anchors in a row and non-rectangular (round, triangular, trapezoidal, L-shaped) anchor configurations.

Considering rock mining in abnormal conditions, e.g., in rescue operations executed in high methane concentrations, or the removal of safety pillars (etc.), mechanical rock mining using specialist machinery and/or the use of explosives is uncalled-for or even prohibited. This, in turn, may necessitate resolving to manual mining methods, whose effectiveness, however, is difficult to assess. With a view to mitigating the risks or uncertainties involved in the application of other alternatives, it appears that harnessing the potential of concrete breakout and concrete cone fracturing mechanisms may prove highly effective.

FEM-3D systems exhibit a good capacity for the analysis of how breakout cones are formed (failure surface) [44,45] and interactions between them, e.g., relative to the effective embedment depth (*h*_ef_) to anchor spacing (*s*) ratio [34,46]. The finite element method has proven its power in supporting fastening techniques—its computational capabilities are, thus, expected to bring considerable benefit to the rock fracture technology. Not only does it enable optimal planning of the layout of anchor holes, so as to produce maximum breakout prisms in given geological and technological conditions, but it also has a bearing on the material removal rate and the energy consumption of mining.

**Figure 2:**
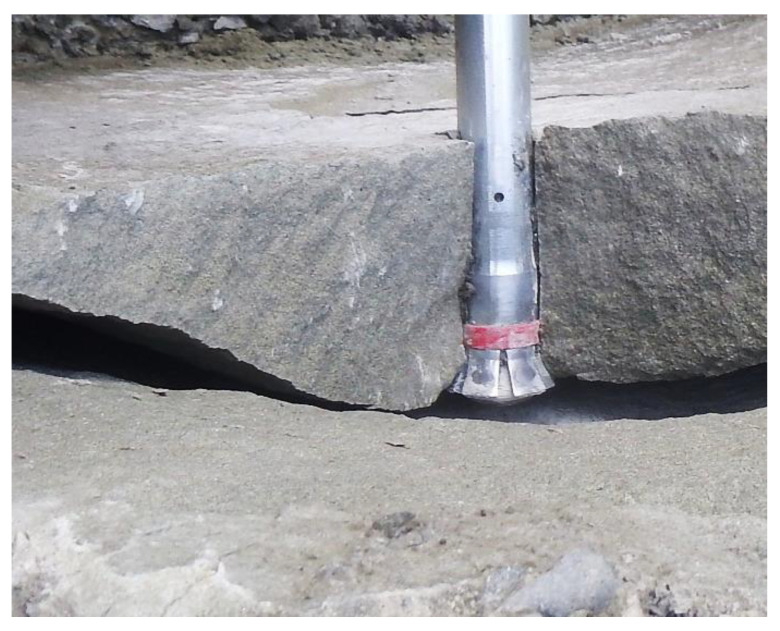
Rock fracture with a single undercut anchor.

**Figure 3:**
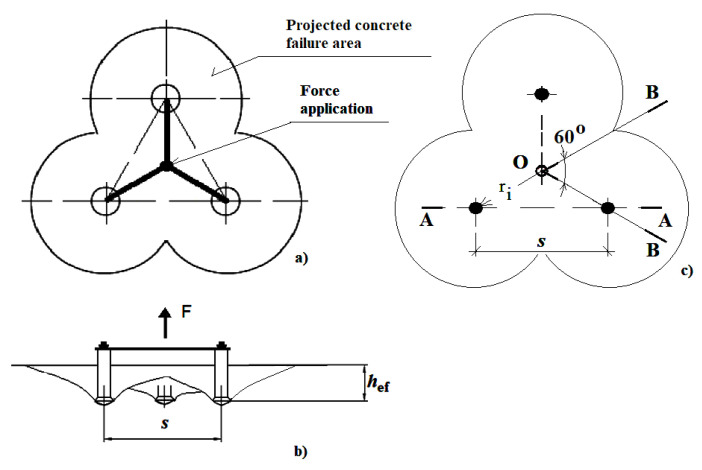
The layout and loading of the 3-anchor system (**a**), ÷ (**b**), (**c**) a BOB section—a sixth of the model serving as the basis for the FEM-3D model of the 3-anchor system, *s*—anchor spacing equal to the 2-anchor system, *r_i_*—radius of anchor spacing.

The key project objective is the study of the fracture mechanism in the aspect of the failure cone size, the respective crack length, and the forces involved in the anchor pull-out from the rock material relative to the layout of the anchors in the anchorage circle, embedment depth, or rock strength characteristics. Considering the group effect in cone failure, the process may be carried out by pulling out successive anchors that are laid out at a specific spacing (*s*)—optimal for their breakout performance. One of the criteria that may serve as a marker of the process efficiency is the volume of the rock mass extracted by a single-anchor or multiple-anchor systems, as it directly translates into the energy consumption of the excavation drilling process or its progress. The factor in question, which is linked to the maximum volume of the breakout prism, is the s/*h*_ef_ ratio. This effect is observed due to the interaction of the failure surfaces that occurs for a given embedment depth, when s < s_gr_ (Figure 3). The group effect enhances the breakout process efficiency: at a given embedment depth, a slightly lower pull-out force converts into a substantial increase in the volume of the prism removed from the rock [47]. The phenomenon is relatively common and occurs, e.g., in machine cutting of brittle material (rock). A rock crack with a single undercut anchor is shown in Figure 2.

Several works have reported on the results from the comparative analysis of single-fastener and two-fastener anchorage systems [44,46,48,49], although 4-fastener systems have been put to pull-out testing as well. In the latter case, however, in-situ testing using a mobile test set-up has highlighted considerable difficulties emerging when the multi-anchor fasteners were to be embedded. An explanation for this may be sought in the highly heterogeneous surface of the rock blocks extracted in the sandstone mining process, which is the source of issues regarding, inter alia, maintaining the same anchoring depth for each of the fasteners. The discrepancies that occur typically lead to the breaking-off of one of the anchors and the loss of stability of the entire fixture—an effective blocker of further testing. Given the limitations, it was resolved that the study was to focus on one-, two-, and three-anchor systems.

During the implementation of the project, extensive research on the mechanical parameters of rocks was conducted. The obtained parameters were fed into FEM numerical analyses using 2D and 3D models. The 2D models used the “cohesive zone” and *K*_Ic_ [48].

The rock parameters were determined from the specimens collected in the field tests using the three-point bending method. *K*_Ic_—in this work, the ASTM formula proposed by Brown and Crawley was adopted. The critical strain energy release rate in fracture mode I was calculated from the formula:

*G*_Ic_ = (*K*_Ic_)^2^ / E [N/m],[N/mm], (1)

E—Young’s modulus and Poisson’s ratio (ν)—during compression tests with the use of extensometers measuring lateral deformations. The compression strength was obtained from the destructive compression test. Fourteen 7 × 7 × 7-cm cubic samples were used to determine material parameters. *K*_Ic_—the stress intensity factor in fracture mode I with three-point bending test [N/mm1.5], ft—tensile strength—the Quasi-Brazilian test [46].

The research conducted to a large extent extends the state of knowledge in the field of fastening technology, used so far in concrete, by the issues of the so-called cone of destruction generated under the action of a set of undercutting anchors fixed in a natural rock medium.

## 2. Model and Material Parameters

This section describes the attempts to estimate the size of the breakout prism and the crack length using the FEM-3D method. In the modeled scenario, the computations were performer for the 3-anchor system of fasteners embedded in the rock mass (Figure 3).

It was assumed that three anchors were simultaneously subjected to the tensile force applied in the center of gravity of the figure determined by the axes of anchors (Figure 3). In practice, the force is applied to anchors by means of a rigid plate with properly spaced anchoring holes.

**Figure 4:**
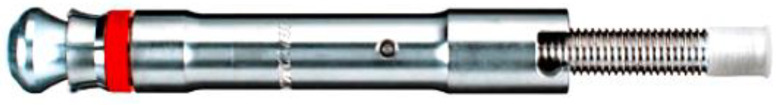
Hilti HDA-P undercut anchor.

The fasteners used in this work were Hilti HDA-P undercut anchors [50]—torque-controlled anchors that cut own undercut by application of setting torque that forces sleeve over cone. Depending on the purpose of simulation, the spacing of anchors or their dimensions were modified within the values set out in HILTI catalogue [50] (M12, M16, or M20) (Figure 4).

In the presented experiment, the modeled base material was sandstone, described by the following parameters described in Table 1 and Table 2.

The criteria specified in the FEM-3D models were damage criterion—“max. principal stress” and damage evolution—“softening linear”; the medium is continuous and homogeneous.

**Figure 5:**
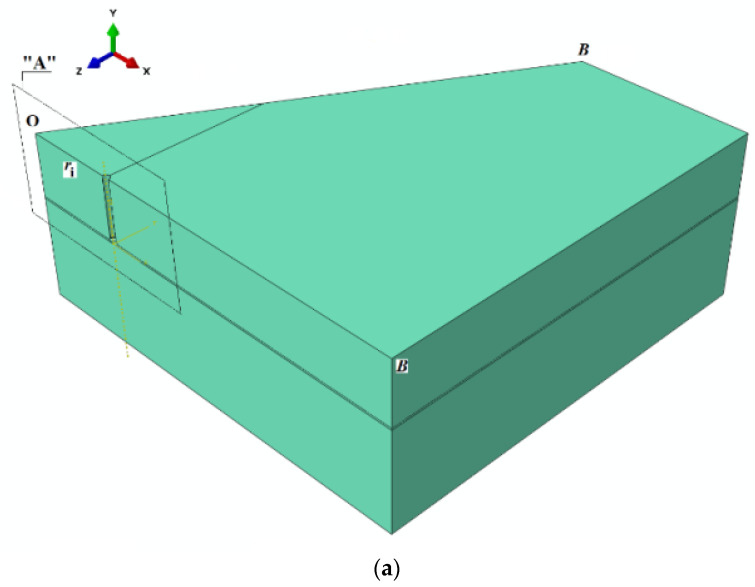

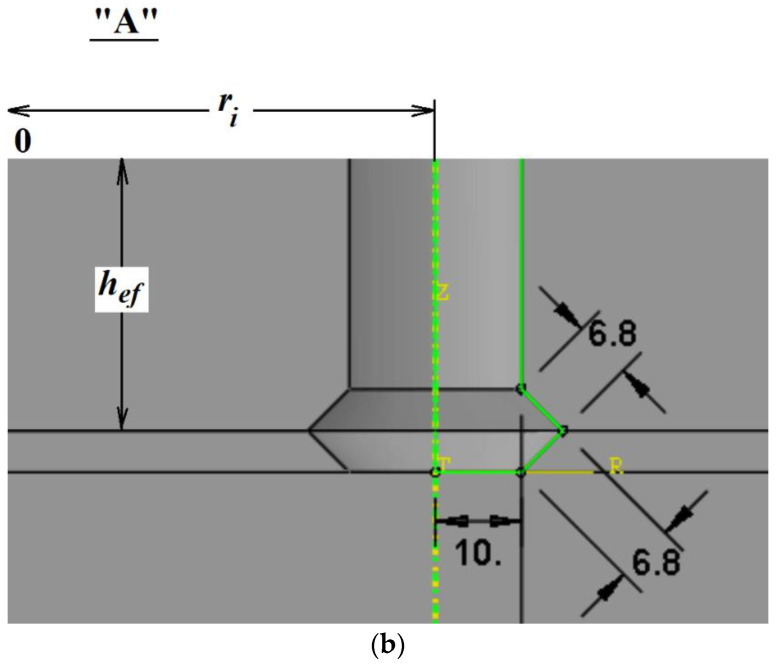
(**a**) The geometric model used in FEM simulation, (**b**) anchor geometry.

The modeled geometry was a prism. Due to the axial symmetry conditions, 1/6 of the mechanical model was used, which was a BOB cross-section area (Figure 3, Figure 5)

The model implements half of the anchor shell profile, as in Figure 5.

The load was applied vertically—along the Y-axis (Figure 5b) to the anchor-rock contact surface, with a maximum value of 10,000 N/mm^2^.

**Figure 6:**
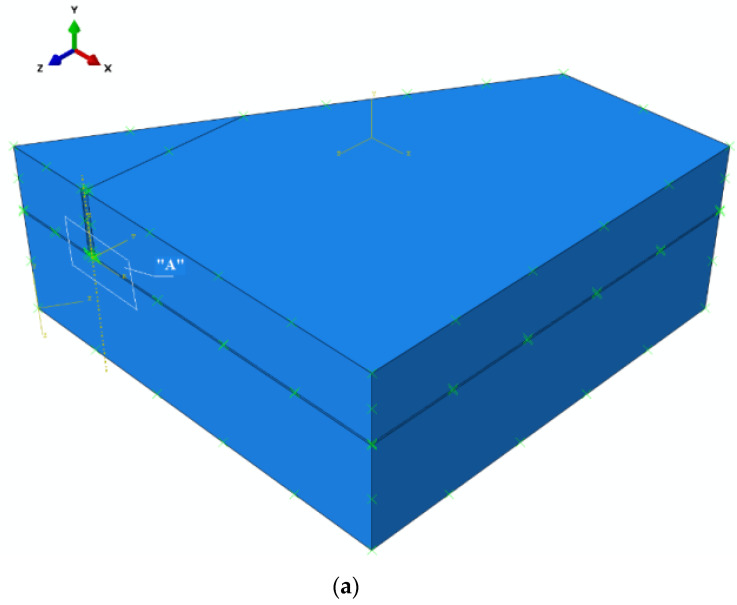

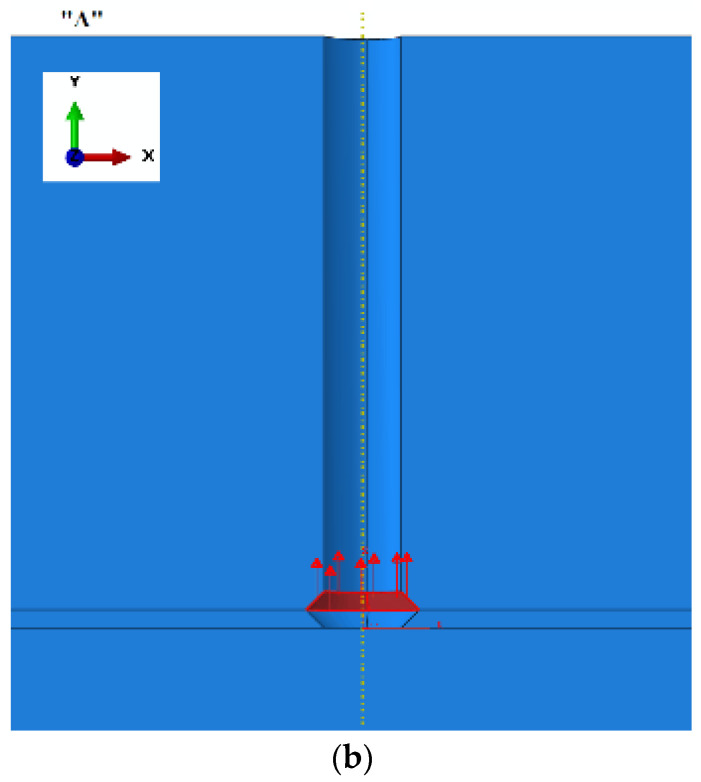
(**a**) Model restraints, (**b**) anchor loading.

Boundary conditions:

Three translational degrees of freedom were used on the base and two perpendicular vertical lateral walls (without the hole) (Figure 6a).

According to the conditions defined in the FEM-3D analysis, the supports have no effect on stress propagation in the failure surface formation zone; as for the material properties, it is considered infinite, continuous, and free from internal structure displacement. Therefore, it is justified to apply boundaries on the nodes in the version proposed for analysis. During field tests, the spacing between fixture points was set so as to reduce to the minimum the effect that the supports could have on the stress distribution in the area of the failure surface.

Mesh parameters:

The numerical analysis was carried out using the eXtended Finite Element Method (XFEM) in ABAQUS (Abaqus 2019, Dassault Systemes Simulia Corporation, Velizy Villacoublay, France).

ABAQUS element: C3D8R—8-node linear brick with reduced integration;

Total number of nodes = 10,848;

Total number of elements = 9229—linear hexahedral elements of type C3D8R.

**Figure 7:**
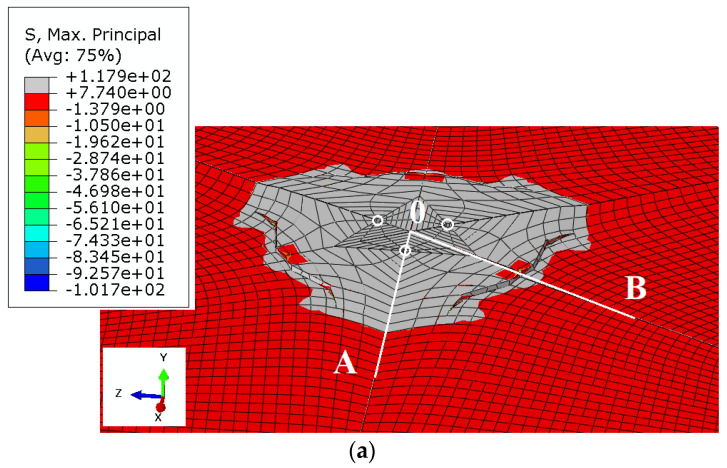

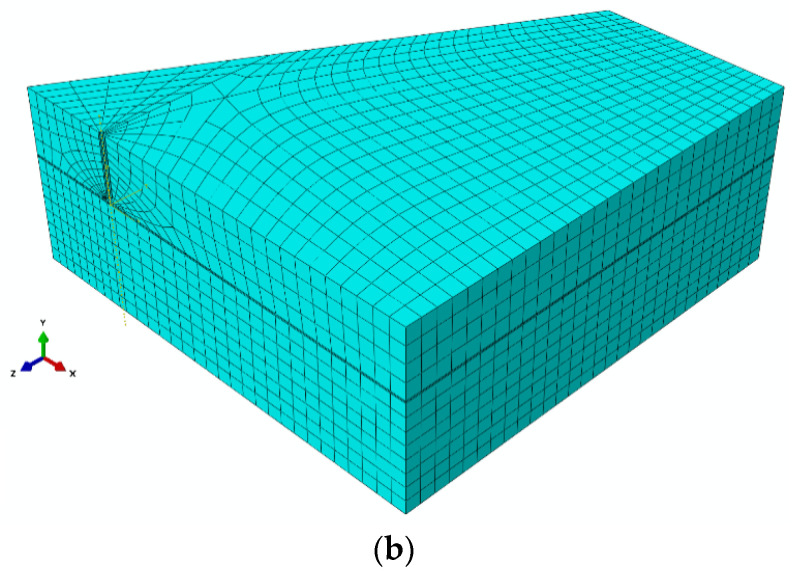
Model discretisation using the Finite Element Method: (**a**) full model, (**b**) the element mesh model obtained from the symmetry in the 3-anchor system.

The mesh was composed of 30 mm elements. The model discretization method (finite element mesh) is illustrated in Figure 7.

The anchor spacing was modified by changing radius *r_i_*. This resulted in the change in the distance between anchor axes *s* in the A-A cross-section (Figure 3), which is equivalent to anchor spacing in the 2-anchor system.

The *r*_i_ radii implemented in the model were (a) 115.47 mm, (b) 230.94 mm, (c) 346.41 mm, and (d) 404.14 mm. Anchor spacing *s* was respectively 200 mm, 400 mm, 600 mm, and 700 mm. Effective embedment depth *h*_ef_ was the same in all test scenarios—150 mm. Therefore, the *s*/*h*_ef_ ratios were 1.33; 2.67; 4.0; 4.66.

**Figure 8:**
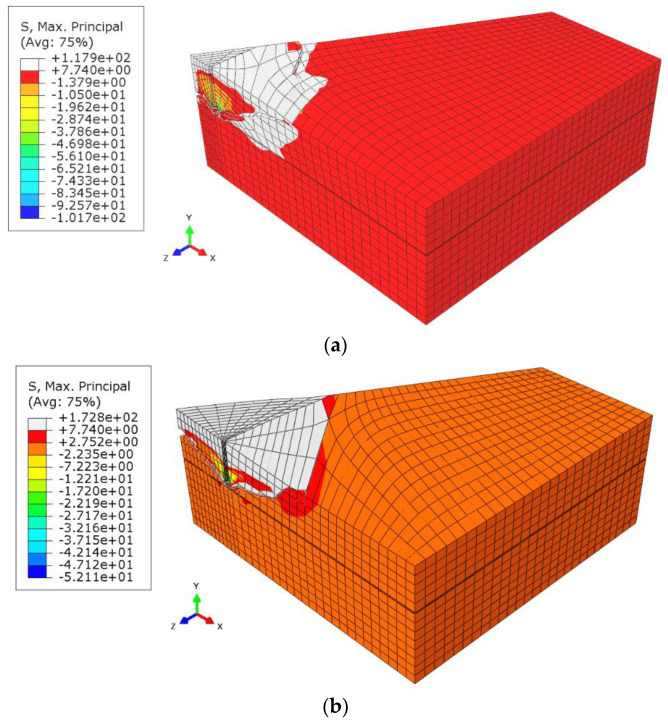

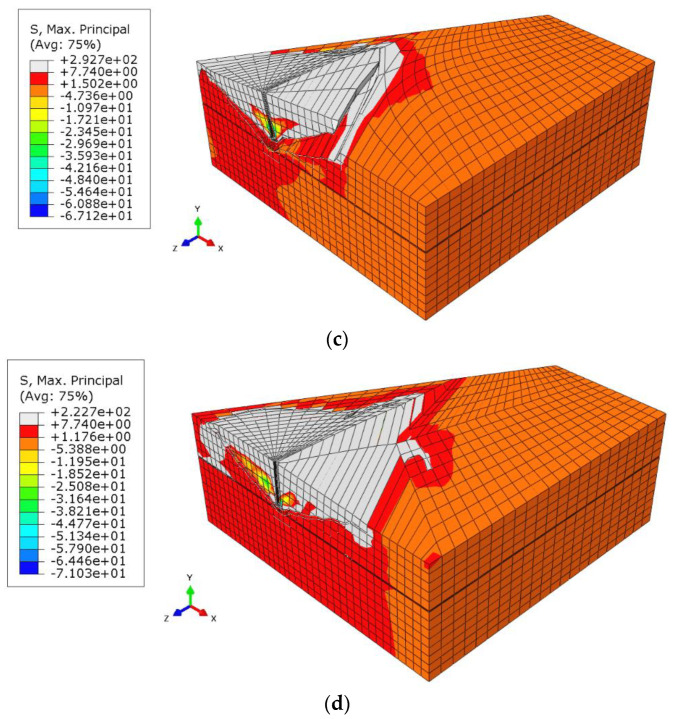
Maximum principal stress distribution and crack propagation in the models (magnified deformations). Radius *r*_i_: (**a**) 115.47 mm, (**b**) 230.94 mm, (**c**) 346.41 mm, (**d**) 404.14 mm. Effective embedment depth *h*_ef_ = 150 mm.

The cone failure in all test cases is shown in Figure 8.

From the simulation results, it can be seen that as the anchor spacing radius *r_i_* increases, the group effect becomes less significant.

**Figure 9:**
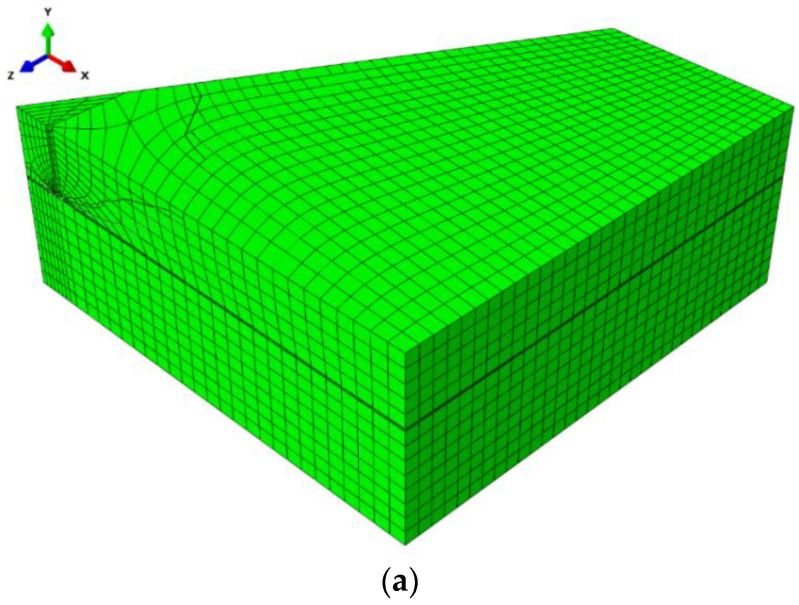

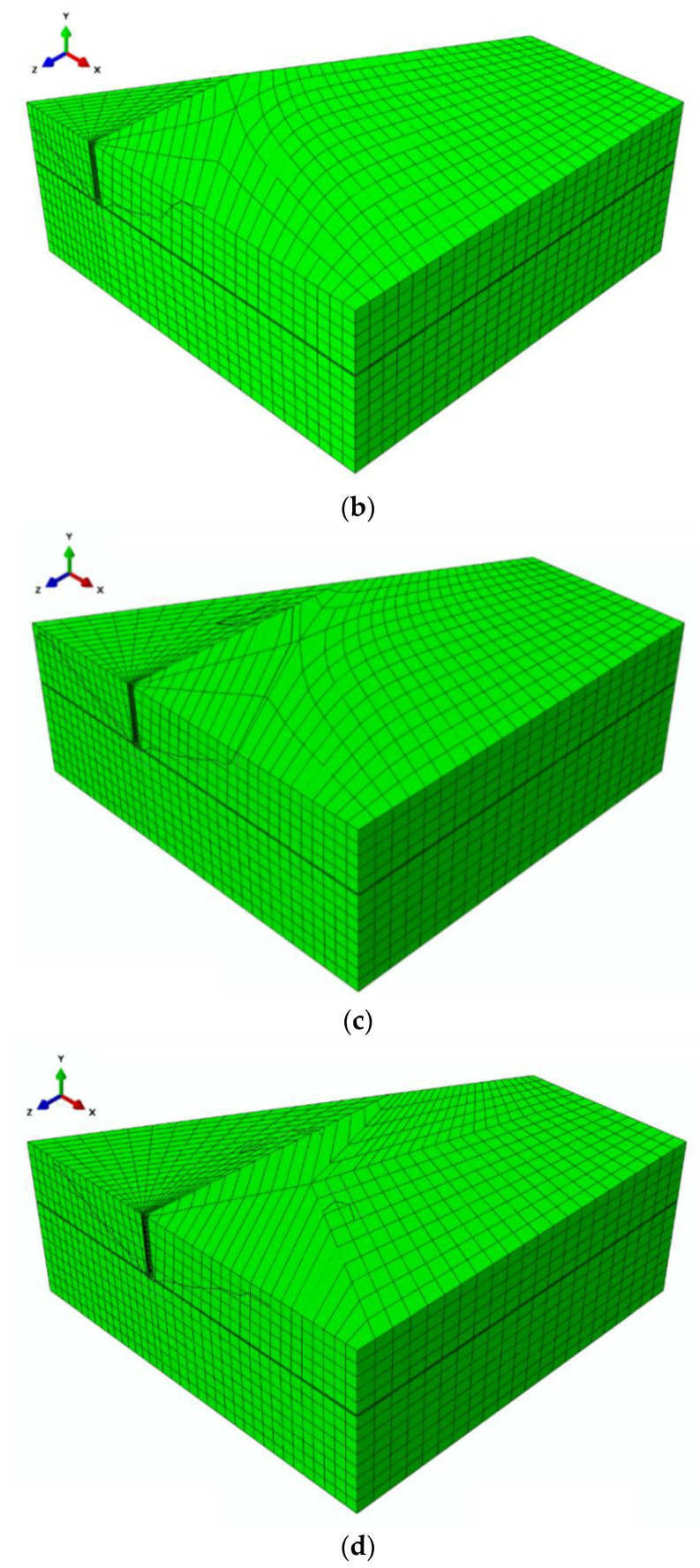
Rock break-out for the 3-anchor system of fasteners, *r*_i_: (**a**) 115.47 mm, (**b**) 230.94 mm, (**c**) 346.41 mm, (**d**) 404.14 mm.

To improve the illustration of the problem, the formation of the failure surface on the mesh without deformations is shown in Figure 9.

A strong group effect was shown to occur for *r*_i_ radius values 115.47 mm and 230.94 mm, whereas it is distinctly weaker for *r_i_* values 346.41 mm and 404.14 mm. The anchor spacing *s* was equal to 200 mm, 400 mm, 600 mm, and 700 mm, respectively. Given that the effective embedment depth *h*_ef_ was 150 mm and the *s*/*h*_ef_ ratios were 1.33; 2.67; 4.0; 4.66, it was demonstrated that the group effect is stronger in the case of *s*/*h*_ef_ ratios 1.33 and 2.67, while for values in the range of 4.0 and in particular 4.66, the group effect is considerably weaker.

**Figure 10:**
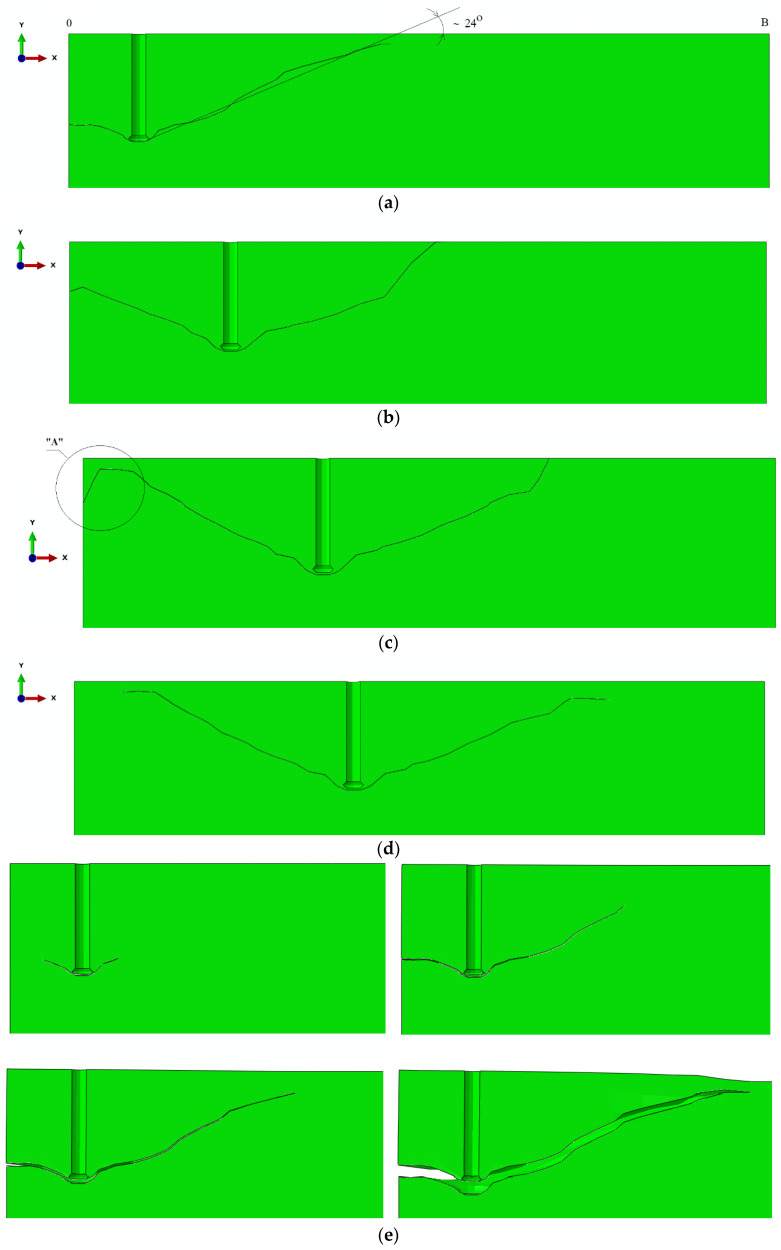
Crack propagation and break-out cone angle *a*, for *r*_i_: (**a**) 115.47 mm, (**b**) 230.94 mm, (**c**) 346.41 mm, (**d**) 404.14 mm. Detail “A”—“wandering” of the gap, (**e**) crack propagation for the anchor system for *r*_i_: 115.47 mm.

In all cases, the value of the angle of the break-out cone in the considered element of the FEM model was on average about 24° (Figure 10).

What emerges from Figure 10 (and Figure 10c in particular) is that in the end-phase of crack propagation, the X-FEM ABAQUS algorithm, governed by the fracture mode I criteria, fails to accurately determine the direction of crack propagation. This, in turn, compromises the accuracy of the determined breakout prism parameters of interest (crack length and the prism angle). It is, thus, necessary to approximate the trajectory, which may lead to various estimation errors.

## 3. Experimental Tests—Verification of FEM Analysis Results

**Figure 11:**
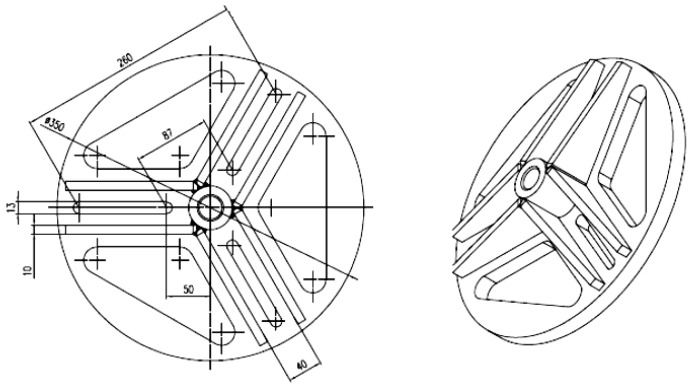
The schematic of the 3-anchor anchorage circle.

The initial assumption was that the group effect occurs between anchors in the anchorage circle, which ensures even distribution of loads to individual anchors. Each test was performed on anchors of the same type and size. Figure 11 shows the anchorage circle for three anchors with the possibility of adjusting anchor spacing.

Each anchor in this group should exhibit the same stiffness (given that they were of the same type, size, and embedment depth). The group effect between anchors loaded with given operating stress occurs if the axial spacing *s* between the anchor axes does not exceed the critical spacing (i.e., according to the CCD procedure, in a system of two anchors, for concrete cone failure ***s*_gr_** = 3***h*_ef_**).

**Figure 12:**
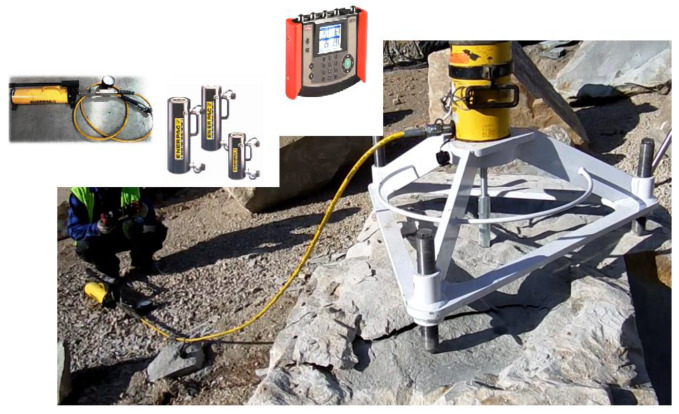
The mobile test stand.

The components of the mobile test stand shown in Figure 12 were

- A support frame for the measuring cylinder with three height-adjustable supports;
- A hydraulic cylinder;
- A hand pump set with a pressure gauge;
- A digital recorder.

The calibration of the measurement path guaranteed that the current force acting on the anchor (calculated from the current pressure in the hydraulic cylinder and the geometrical parameters of its components) is precisely assessed.

**Figure 13:**
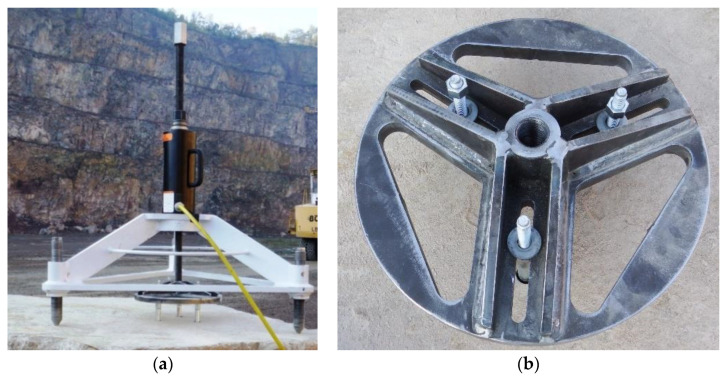
(**a**) Testing instrumentation for the 3-anchor pull-out tests, (**b**) the 3-anchor anchorage circle.

The frame with the testing actuator and the three-anchor anchorage circle are presented in Figure 13a, and the latter is shown separately in Figure 13b.

The tests were conducted at four distinct locations, “Zalas”, “Braciszów”, “Guido”, and “Brenna”, exhibiting profound differences in terms of rock structure and strength parameters. Table 3 shows the mechanical characteristics of rocks in individual mines.

**Figure 14:**
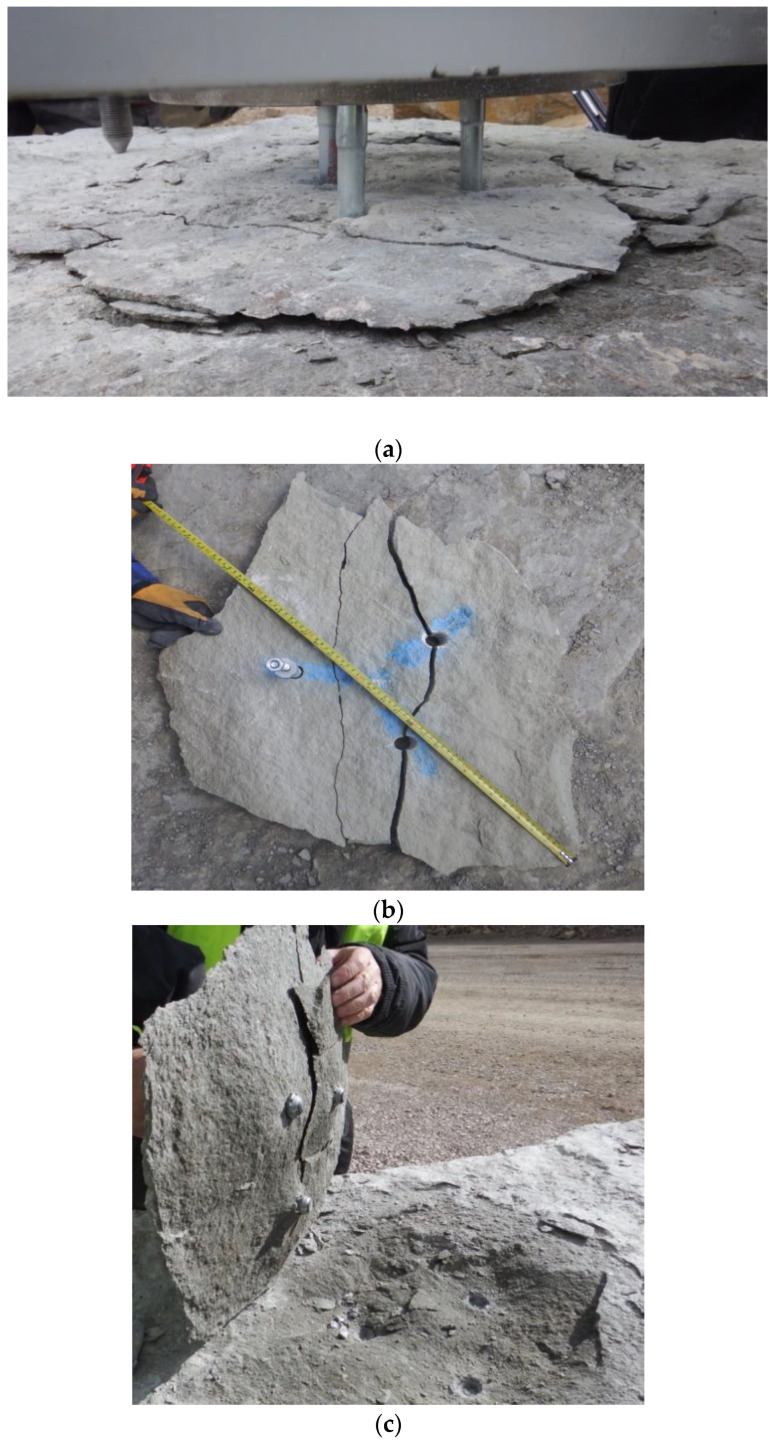

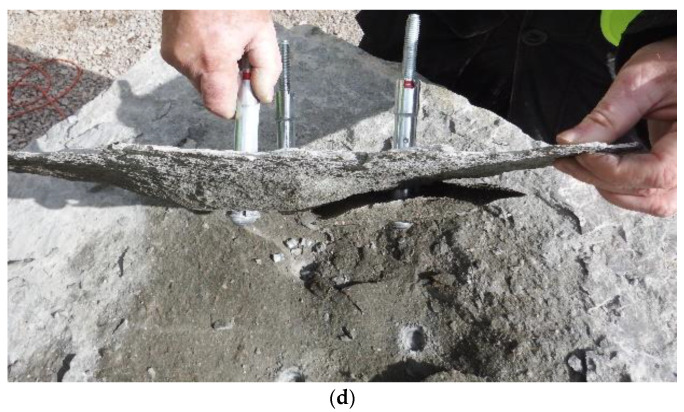
Break-out prisms—the 3-anchor group: (**a**) fractured rock material, (**b**) failure surface, (**c**)–(**d**) group effect in rock cone failure (a group of 3 anchors).

The breakout prism specimens produced as a result of tensile loading of three-anchor anchorage circles are presented in Figure 14. In the several dozen satisfactory tests, the rock slabs showed certain variety with respect to their internal structure and strength parameters, as well as the results: the values of the pull-out force, crack propagation length, and the shape of the failure surface.

The failure surfaces of the breakout prisms were scanned and digitalized to an image for further assessment of their characteristic parameters.

The failure surface was scanned using a 3D digital laser scanner. The point cloud obtained from the scan was manually processed and converted into an STL triangulated surface. Subsequently, a specialist *LEIOS 2* R10 software (E.G.S. Srl, 2019, Bologna, Italy,) was used to process and convert the STL model into the *.sat model. The 3D solid model of the failure surface after the breakout failure was subsequently processed with the use of the *Inventor* software to obtain a derivative element in the form of a pull-out block.

**Figure 15:**
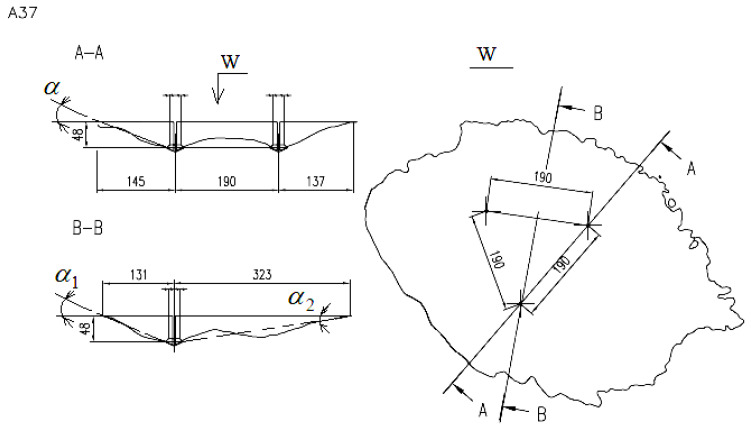
An outline of the failure surface on the free surface of the rock material and cross-sections of a break-out prism pulled out by the 3-anchor anchorage system.

The 3D solid model provided data on the failure surface area, as well as the size and shape of the surface of the breakout prism base or its volume. The failure surface obtained from 3D scanning can be viewed from any perspective, thus the crack trajectory is accessible in any cross-section, which, for a group of three anchors, is shown in Figure 15.

Figure 15 shows an outline of a break-out prism and its cross-sections through two adjacent anchors (cross-section A-A, Figure 15) or through the anchor axis and the center of the anchorage circle (cross-section B-B). Angles *α*, *α*_1_, and *α*_2_ are the failure cone angles in cross-sections A-A and B-B.

For the tested embedment depth, the average angle of the failure cone *α* in the A-A section was ~15°. In the B-B section, the angle *α*_1_ was of the order of 16° and the value of *α*_2_ was on average around 9°.

**Figure 16:**
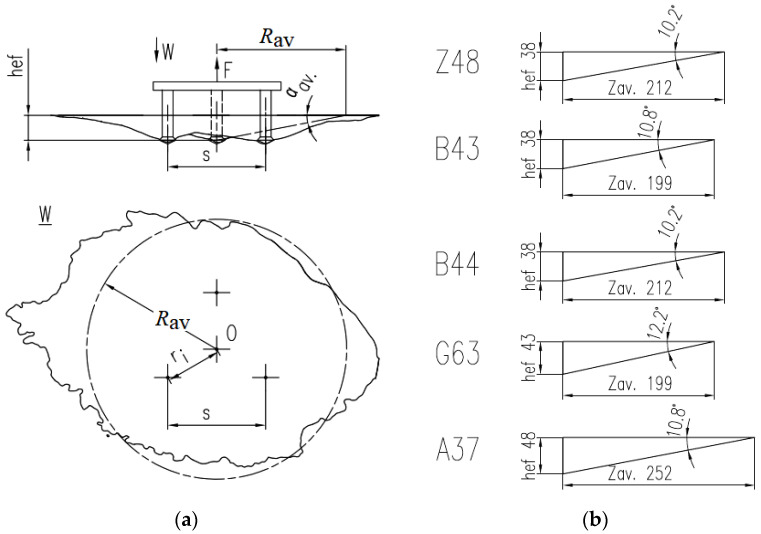
Mean failure cone parameters for a group of 3 anchors (**a**), mean failure cone angles measured in subsequent breakout tests (**b**). *R*_av_—failure cone base diameter, *α*_av_—mean failure cone angle.

Considering the large discrepancies between the prism cross-sections, the sizes of the failure cones, and the resulting ambiguity, it was resolved that an averaged model of a break-out should be determined and adopted for further computations (Figure 16).

The results are presented in Table 4.

The test codes in the first column of Table 4 describe particular test cases. The first letter designates the mine: Z—Zalas, B—Braciszów, G—Guido, B—Brenna. The number is the number of a test performed in a given mine. The measurement documentation for each measurement includes additional specific data: changes in the anchor pull-out force values during testing, effective embedment depth, laser scans of the failure surface, rock morphology, and strength parameters.

As can be seen from the data above, in this case, the mean breakout cone angle was in the range of 10° to 12°. The methodology applied in this study may be regarded as a reliable predictor of the breakout cone angle formed in the pull-out tests of the three-anchor fastening system.

## 4. Discussion

### 4.1. Data Collection and Analysis

The problem presented in this report is analyzed according to the current state of knowledge and the scope of this work. Commonly, the material is considered as a linear-elastic, continuous, and homogeneous medium. The problem of rock/cement breakout using mechanically fixed anchors is approached as the fracture mode I of linear-elastic fracture mechanics. These assumptions are reflected in the computations and the existing models of concrete breakout for anchorages. Therefore, the premise of the study was to investigate the behavior of a natural rock mass during the breakout process performed using undercut anchors loaded in tension.

The assessment of the degree of correlation between the results from the numerical analysis and the industrial research must essentially account for the fact that the numerical model assumes the continuity and homogeneity of the rock material described by the particular mechanical parameters, e.g., compression strength, fracture energy, Young’s modulus, Poisson number, cohesion, and angle of internal friction. For natural reasons, in certain cases, there are distinct discrepancies between the results from in-situ measurements. The test material for testing was extracted from distinct locations, which were characterized by different strength parameters, internal structure, and moisture content. Randomly distributed artefacts, including RQD or discontinuities, were found in the internal structure of the rock [51,52,53]. The scans of the breakout cone surface revealed characteristic crack propagation trajectories. In the homogeneous material, the crack was shown to propagate symmetrically to the anchor axis (in the single-anchor system) or to the symmetry planes of the multiple-anchor systems. Due to the presence of the artefacts in the rock material, the crack propagation and the shape of the breakout prism depend on the local structure of rock. Another aspect to consider is the coefficient of sliding friction of rock on the anchor head. The unfavorable and variable conditions in the field tests, such as inter alia, the presence of moisture in the rock-anchor system, impaired the precision of measurements. Therefore, the numerical analyses were performed for a number of friction coefficient variants in order to obtain the result that would enable, in the following stages, to calibrate the FEM model (both [48,49] and 3D [44,46]). In the case when the crack propagation can be determined with little ambiguity, the 3D and 2D results exhibit high consistency [48,49]. For reasons of space and the scope of research objectives, only one of the analyzed cases is presented in the article. Including further detailed examples would require substantial analytical works involving the comparisons of the fracture trajectories in matching cross-sections as well as in a clear homogeneity of the rock material (as per the FEM model assumptions). This can be easily identified by the symmetry of the failure surface/crack trajectory obtained from 3D scans of the failure surface produced in breakout in tension. Moreover, the numerical models and actual breakout prism specimens selected for a comparative analysis must have equal values of the coefficient of sliding friction. This assumption is difficult to follow in practice, however, due to the vast differences between rock specimens in terms of moisture or grain size, even within the same rock group (especially in sandstones). Considering the variability of the end-phase crack propagation in the FEM analysis with the use of the ABAQUS system algorithm, the computations were halted prior to the cracks’ reaching the free surface. In each case, the moment when the calculations were stopped was different. Most importantly, the results from the crucial part of the calculations, i.e., in the vicinity of the critical crack opening (at 0.35–0.45 of the crack length measured along the lateral surface of the failure cone), are valid and eligible for the comparative analysis. In this regard, the applicability of fracture mode I is beyond a reasonable doubt.

After the comparison criteria have been fulfilled, the numerical and field test results displayed a satisfactory level of compatibility with respect to the research goals of the project. Considering the substantial scatter of results, which has been attributed to the variable field testing conditions, mean breakout prism angle values were determined for individual measurement clusters. The approach to data collection and analysis ensured that the obtained results are a trustworthy point of reference for further estimation of the research project parameters carried out in the next stage of industrial research, taking into account the group effect in cone failure.

### 4.2. Results

Angle *α*_1_ in the cross-section B-B (Figure 15) corresponds to the crack propagation angle obtained from the 3D-FEM models (Figure 10). The results indicate that the crack propagation angle from in-situ experiments was notably lower than predicted by the FEM models.

The Finite Element simulations of failure cone angles produced by the pull-out of one-anchor [44] and two-anchor anchorages [46] are comparable and in agreement with the literature data [54] (the angle from the FEM model was around 22°). Studies have shown [29] that for low embedment depths in concrete (similar to those used in the research project), the crack propagation angle is about 28°. It would then seem that FEM models can be adjusted to obtain results convergent with experimental tests. The discrepancies concerning simulations of rock (sandstone) parameters and fracture could be explained by the problems with a correct estimation (even in laboratory conditions) of critical fracture energy rate G_fc_—a quantity that is central to the FEM ABAQUS system computations.

The results emerging from this study slightly differ from our previous findings (e.g., [44,46,48,49]). The disparities are observed both in terms of the failure surface dimensions and the prism angle. In the latter case, for instance, the mean angle was 24° whereas in our past findings 25° [44]. The discrepancies result from the limitations of the FEM ABAQUS tool, which was incapable of simulating the end-phase crack propagation in rock failure. It was, therefore, necessary to allow for the mixed fracture mode in order to improve the fit of numerical and experimental results. Similar issues have been reported by other researchers [44,46].

The results from simulations have been found to depend strongly on other assumptions, such as the coefficient of sliding friction of rock on the anchor head, Young’s modulus, and critical fracture energy rate (G*_f_*_c_) [48]. The coefficient of friction is a major influence on the propagation and length of crack leading to rock fracture. In specific terms, in our previous findings [44,46], it was established that the coefficient of friction of 0.2 allowed for successful calibration of the FEM model considering the shape and dimensions of the failure surface and the values of the breakout force.

In-situ sandstone testing has shown that the most considerable differences in the value of failure cone angles were exhibited with respect to the norms and rules for concrete. The CCD procedure assumes a failure cone angle of 35°, however, it emerges from our field tests of three-anchor groups that the angle oscillated between 10° and 12° (according to the employed mean failure cone angle assumption).

It ought to be recapitulated that in CCD [14] the degree of approximation used in failure surface estimation is so high due to the fact that it only concerns a part of the crack trajectory, i.e., to the point where it amounts to the critical value. Afterwards, in the post-critical stage, the crack propagates abruptly, involving minimum force. This explains why crack propagation in the post-critical phase is of marginal importance for the load-carrying capacity of the anchor. It is also the reason why a certain degree of approximation is acceptable. It does, however, strongly affect the estimation of the volumetric efficiency of the anchorage pull-out technology.

## 5. Conclusions

The reported study concerned rock mining in extreme conditions (e.g., methane hazard, geological and mining restrictions). It was an attempt to determine whether the current procedures and recommendations, set out in the respective international standards for anchorage in concrete, are a reliable point of reference for the purpose of determining the undercut anchor pull-out parameters. A further motivation was to broaden the state of the art for the potential application of anchorage technology to fasten structural components in a natural rock mass.

The findings from this study have shown that the common approximations currently employed in the problems of material fracture as a result of undercut anchor pull-out based on fracture mode I provide an insufficient explanation for the breakout mechanism and the estimation of the failure surface dimensions resulting from the undercut anchor pull-out procedure. The crack trajectories recorded in the field tests as well as the variable results obtained in the estimation of the crack propagation direction at the vertex of the crack using the fracture mode I-based ABAQUS system algorithm necessitated the use the mixed fracture case (Mode I + II).

Despite including the approximations in the determination of breakout prism angles, both with respect to the results from the in-situ tests and the numerical computations, our results display substantial differences in the trajectory and length of crack propagating through the rock medium compared to the CCD standard approach and recommendations for concrete. Therefore, the current knowledge in the field of undercut anchors technology, considering the fastening methods and the proposed method for controlled fracture of the rock medium using undercut anchors, cannot be directly applied in engineering practice.

Both the 3D-FEM analyses and the experimental tests conducted as part of this study demonstrate that considering the testing/simulation conditions applied in the tests—embedment depth, sandstone—the size of the breakout prism and the crack length is notably higher than it results from the calculation procedures contained in CCD recommendations. The experiment shows that in a cross-section through two anchors (Figure 15, A-A), the *s*/*h*_ef_ ratio is, in the majority of cases, in the range of approx. 3.6–4.0. The parameter is, thus, shown to exceed the recommendations following from the CCD method, which specify that its maximum value cannot be higher than 3.0; the obtained results are then 20–33% higher than the standard CCD values. Furthermore, the numerical results indicate that for the nominal breakout prism angle, the critical *s*/*h*_ef_ ratio for which the anchor group effect occurs is ~4.5 (the A-A cross-section (Figure 3) corresponding to spacing in the two-anchor system).

It should be emphasized, however, that the CCD method is dedicated to concrete, whilst in the case of rocks, there is a distinct paucity of relevant scientific data.

In the considered cross-sections, the failure cone angles reported from the FEM analyses and experimental tests (Figure 15) are significantly smaller than recommended by the CCD method. The angle value estimated by the CCD method is ~35°, while from the FEM analysis approx. ~24° (depending on the simulation conditions, including in particular embedment depth, rock strength parameters, or Poisson’s ratio). Nevertheless, it has emerged from the experimental part of the study that the value of the angle in question is even smaller and fluctuates around an average of 15°, frequently dropping to the ~10–12° range of values in the mean failure cone for the three-anchor group of fasteners.

The obtained results carry significant implications for planning the outlay of anchor holes in the rock fracture technique using the postulated method.

Given the inherent nature of rocks, characterized by high heterogeneity of geological structure (including extensive cracking and fracturing), the presented results need to be approached as estimates. At the same time, the future investigations must aim to increase the precision of the predicted effects of technological conditions of multi-fastener anchorage pull-out technique.

## Figures and Tables

**Table 1:** Material parameters.

Material Model	Linear Elastic
Young’s modulus (E)	14.276 MPa
Poisson’s ratio (ν)	0.247
Tensile strength (*f*_t_)	7.74 MPa
Damage criterion	“max. principal stress”
Damage evolution	type Energy, Softening: linear
Critical fracture energy rate (G_fc_)	0.335 N/mm
Damage stabilization	Cohesive with viscosity coefficient = 1 × 10^−6^

**Table 2:** Model geometry.

Length	1000 mm
Width	1000 mm
Height	400 mm
Model outline angle (BOB section)	600

**Table 3:** Mechanical parameters of the studied rocks.

Mine	f_c_ (MPa)	Standard Deviation	f_t_ (MPa)	Standard Deviation	k = f_c_/f_t_	ϕ (°)	C (MPa)	Rock	Description
Zalas	106.5	23.86	5.9	1.91	18.1	54	8.6	porphyry	Deck strongly undulating
Braciszów	155.3	29.17	8	0.64	19.41	49.5	14.5	sandstone	Sandstone strong, compact
Brenna	58.8	9.29	3.9	1.17	15.1	53	6	sandstone	Sandstone layered, weak
Guido	97.4	25.52	6.2	0.94	15.7	49.6	11.9	sandstone	Sandstone compact, medium strength

*f*_c_—compressive strength, *f*_t_—tensile strength, *c*—cohesion, *ϕ*—angle of internal friction, k—strength asymmetry factor.

**Table 4:** Anchor group—3 anchors Hilti M12. Parameters and test results.

Test No.	*h*_ef_ [mm]	*s* [mm]	*r*_i_ [mm]	*s*/*h_e_*_f_	*r*_i_/*h*_ef_	*F* [kN]	*R*_av_.	*α*_av_	*V* [dm^3^]
Z48	38	135	78	3.6	2.1	155.86	212	10.2	2.08
B43	38	125	72	3.3	1.9	148.19	199	10.8	2.68
B44	38	140	81	3.7	2.1	204.40	212	10.2	3.21
G63	43	125	72	2.9	1.7	206.96	199	12.2	3.22
A37	48	190	110	4.0	2.3	114.98	252	10.8	4.00

*V*—a volume of the break-out prism, *F*—a value of the break-out force, *s*—a distance between 2 anchors (anchor spacing), *h*_ef_—effective embedment depth, *r*_i_ radius of anchor spacing.
